# Identification of susceptibility genes in non-syndromic cleft lip with or without cleft palate using whole-exome sequencing

**DOI:** 10.4317/medoral.20758

**Published:** 2015-10-09

**Authors:** Ya-Peng Liu, Li-Fang Xu, Qi Wang, Xiao-Long Zhou, Ji-Long Zhou, Chen Pan, Jin-Peng Zhang, Qin-Rong Wu, Yi-Qun Li, Yu-Juan Xia, Xiu Peng, Mei-Rong Zhang, Hong-Min Yu, Li-Chun Xu

**Affiliations:** 1 School of Public Health, Xuzhou Medical College, Xuzhou, Jiangsu 221004, China; 2 Obstetrical Department, Maternal and Child Health Hospital of Xuzhou City, Xuzhou, Jiangsu 221009, China

## Abstract

**Background:**

Non-syndromic cleft lip with or without cleft palate (NSCL/P) is among the most common congenital malformations. The etiology of NSCL/P remains poorly characterized owing to its complex genetic heterogeneity. The objective of this study was to identify genetic variants that increase susceptibility to NSCL/P.

**Material and Methods:**

Whole-exome sequencing (WES) was performed in 8 fetuses with NSCL/P in China. Bioinformatics analysis was performed using commercially available software. Variants detected by WES were validated by Sanger sequencing.

**Results:**

By filtering out synonymous variants in exons, we identified average 8575 nonsynonymous single nucleotide variants (SNVs). We subsequently compared the SNVs against public databases including NCBI dbSNP build 135 and 1000 Genomes Project and obtained an average of 203 SNVs. Total 12 reported candidate genes were verified by Sanger sequencing. Sanger sequencing also confirmed 16 novel SNVs shared by two or more samples.

**Conclusions:**

We have found and confirmed 16 susceptibility genes responsible for NSCL/P, which may play important role in the etiology of NSCL/P. The susceptibility genes identified in this study will not only be useful in revealing the etiology of NSCL/P but also in diagnosis and treatment of the patients with NSCL/P.

**Key words:**Non-syndromic cleft lip with or without cleft palate, whole-exome sequencing, sanger sequencing, susceptibility gene, single nucleotide variants (SNVs).

## Introduction

Non-syndromic cleft lip with or without cleft palate (NSCL/P) is one of the most common human birth defects in all populations worldwide. The average birth prevalence of NSCL/P is estimated to be 1/700 ranging from 1/500 to 1/1,000, depending on the economic, geographical, and genetic background variation (1). Individuals with NSCL/P may experience problems with feeding, digesting, speaking, hearing and social engagement which can be corrected to varying degrees by multiple rounds of surgical repair along with dental treatment, speech therapy and psycho social intervention from birth until adulthood. Furthermore, affected children have higher morbidity and mortality throughout life than do unaffected individuals (2). Therefore, NSCL/P will bring substantial psycho social and financial burdens to individuals, their families, and society, which creates a major social problem (3).

The etiology of NSCL/P has been a focus area of research for centuries. The etiology of NSCL/P is complex and is currently considered to be due to a combination of both environmental and genetic factors (4), which is distinct from that of syndromic CL/P (SCL/P). SCL/P has been shown to associate with single-gene mutations, chromosomopathies, and exposure to teratogens (5). However, the etiology of NSCL/P remains unknown because of its etiologic complexity (6). Furthermore, NSCL/P is the most prevalent type of CL/P and approximately 70% of cases are considered to be NSCL/P (7,8). Therefore, more studies are necessary to elucidate genetic risk factors related to NSCL/P. To date, approaches to uncover genetic determinants controlling risk of NSCL/P include linkage analysis, association studies, identification of chromosomal anomalies or micro deletions in cases, and direct sequencing of DNA samples from affected individuals (9). Various loci could have a causal role in NSCL/P including certain regions of chromosomes 1, 2, 4, 6, 14, 17, and 19 in previous human studies (10,11). Variants of candidate genes linked to NSCL/P including TGF-α, TGF-β3, MSX1, IRF6, TBX22, CYP1A1, MTHFR, and RARA have been identified in population-based studies (1,4,12-14). Although many genes and regions of chromosomes have positive results in one or more of the studies, few of those findings were consistently positive across all studies.

The methods to genotype all single nucleotide polymorphisms (SNPs) using genotyping arrays greatly facilitate the development and progress of genome-wide association studies (GWAS) (15) . GWAS have become a powerful and ideal technique in studying complex human diseases. To date, there have been a number of GWAS for CL/P (16-19). The results of the GWAS are encouraging. GWAS are novel and rapidly advancing approach in the study of molecular genetics underlying human diseases and have led to many exciting discoveries. However, traditional array-based GWAS primarily test the common disease–common variant hypothesis (15). Therefore, GWAS are limited to known and common variants. Only a fraction of the genetic basis of common diseases can currently be explained by associations with common variants (20). Uncommon and rare variants may play a major role in many phenotypes.

Recently, next-generation sequencing (NGS) technology has developed rapidly and has been suggested as a reliable method for finding variations associated with various disease states. NGS technology enables researchers to interrogate the complete human genome or exome for the detection of both common and rare variants, hence improving the chance of finding disease causal variants (21). Whole-exome sequencing (WES) is both a cost-effective and a time-efficient alternative to whole-genome sequencing, so the method is becoming a popular NGS strategy by capturing and sequencing the ~1% of the human genome coding for protein sequences. Ng SB and colleagues first showed that WES can be used to identify disease genes in 2009 (20). Now, WES has been successfully utilized to identify the causative genetic variants responsible for Mendelian disorders and complex hereditary diseases (22,23). One recent study has conducted a WES study to search for potentially causal variants using affected relatives drawn from multiplex cleft families (24). The oral cleft families include two of Chinese origin (one each from Taiwan and Shanghai) with four subjects.

In this study, we conducted WES in eight fetuses with NSCL/P in China. The purpose of this study was to illuminate the susceptibility genes related to NSCL/P.

## Material and Methods

-Subjects and samples

Eight fetuses with NSCL/P were recruited from Maternal and Child Health Hospital of Xuzhou, Jiangsu, China. The cases of NSCL/P were diagnosed by prenatal B-ultrasonography and confirmed by autopsy. The parents of the fetuses were informed of the purpose of the study. The signed informed consent sheets were obtained from the parents of all study participants. After obtaining informed consent, the lip tissue specimens of the fetuses were obtained by sectioning the lip tissue around the cleft of the lip. The study was approved by Ethical Committee for Human Research of Xuzhou Medical College.

-DNA preparation and library construction

Genomic DNA was extracted from the tissues using QIAamp DNA Mini Kit (Qiagen, Valencia, CA, USA) according to the manufacturer’s protocols. The quality of the DNA sample was ascertained by electrophoresis and determined to be of high molecular weight with no visible degradation. Quantity was determined using standard Pico Green assays. A high quality DNA was used as the starting material. The DNA was fragmented by Bioruptor Sonicator (Diagenode, USA). High-quality DNA library was constructed from genomic DNA using the TruSeq DNA Sample Preparation Kit for end repair, dA tailing, adaptor ligation and DNA fragment enrichment.

-Exome capture and Illumina exome sequencing

The TruSeq Exome Enrichment Kit was used to capture exome sequences from the prepared human DNA library. After two rounds hybridization and washing, the DNA exome library was captured and enriched. The size was checked using a DNA specific chip, the Agilent DNA 1000 on Agilent Technologies 2100 Bioanalyzer (Agilent Technologies, Santa Clara, CA). The libraries were quantified using Qubit (Invitrogen, CA, USA) according to the manufacturer’s instructions. All sequencing of post-enrichment shotgun libraries was carried out on the Illumina HiSeq 2000 Analyzers for 90 cycles per read following the manufacturer’s protocols and using the standard sequencing primer.

-Data filtering and bioinformatics analysis 

After the entire run was completed, image analyses, error estimation and base calling were performed using the Illumina Pipeline (version 1.3.4) to generate primary data. Indexed primers were used to identify the different reads from different samples in the primary data. Only reads that were perfectly matched to the theoretical adapter indexed sequences and reads that matched the theoretical primer indexed sequences with a maximum of three mismatches were considered to be acceptable reads. The local dynamic programming algorithm was used to remove a few unqualified sequences from the primary data using.

**Figure 1:**
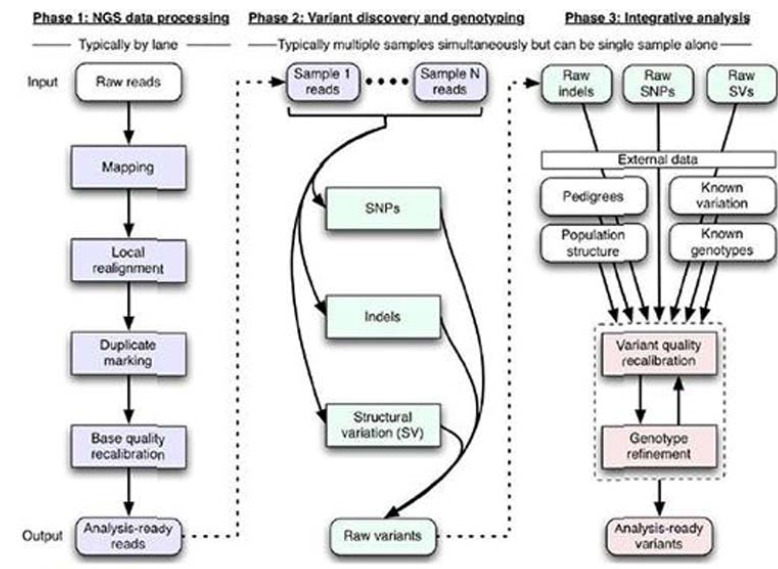
Variants analysis strategy for NSCL/P using GATK tools. Data analysis process was carried out according to step 1-4 including initial mapping, refinement of the initial reads, multi-sample indel and SNP calling and quality score recalibration in phase 1-3 including NGS data processing, variants discovery and genetyping and integrative analysis. The SNPs in the exomes were identified.

After filtering, for aligning the sequences to the human genome, the Burrows-Wheeler Aligner (BWA) was used to map short-reads from fastq files against the human reference genome. Then, the Genome Analysis Toolkit 1.6 (GATK) was applied to carry out regional realignment and quality score recalibration and Picardwas applied to mark duplicates. Variants were called using the GATK variant calling program (Fig. 1). The ANNOVAR (Annotate Variation) was used to annotate possible mutation candidates. The variants were compared with NCBI dbSNP 135 and 1000 Genomes Project reported SNP to remove the published variants.

-Sanger sequencing

The Sanger DNA sequencing method has been considered to represent the gold standard for screening variants of genes of interest in many scientific fields. In this study, to verify the DNA sequence variants detected by WES, the variants of the affected individuals were sequenced using Sanger sequencing. The primers were designed for the target region using Primer 6.0. All exons of selected genes were amplified. The PCR products were sequenced on an ABI 3730 DNA analyzer following standard procedures (Life Technologies, USA). The sequence reads were analyzed using the Sequencher software package (Gene Codes Inc, USA). All sequence traces were manually reviewed to ensure the reliability of the genotype calls. The sequencing traces were visually inspected in Finch TV v1.4 (Geospiza Inc, USA).

## Results

A strategy of WES by hybrid capture was employed ( Table 1). The average total effective data were 12.4 billion base pairs (Gb). The average ratio of reads alignment to reference genome was 94.2%. On average, the efficiency of the hybrid capture was 60.7%. When measured at a minimum depth of 4×, 93.1% of the target region was covered. Likewise, when measured at 10× and 20×, 90.5% and 86.1% of the intended target was covered, respectively. The average mean coverage sequencing depth on official target was 101.1-fold and with 95.8% fraction of official target covered. Thus, the depth and coverage should be adequate to reliably detect DNA variants within the majority of the targeted regions.

**Table 1:**
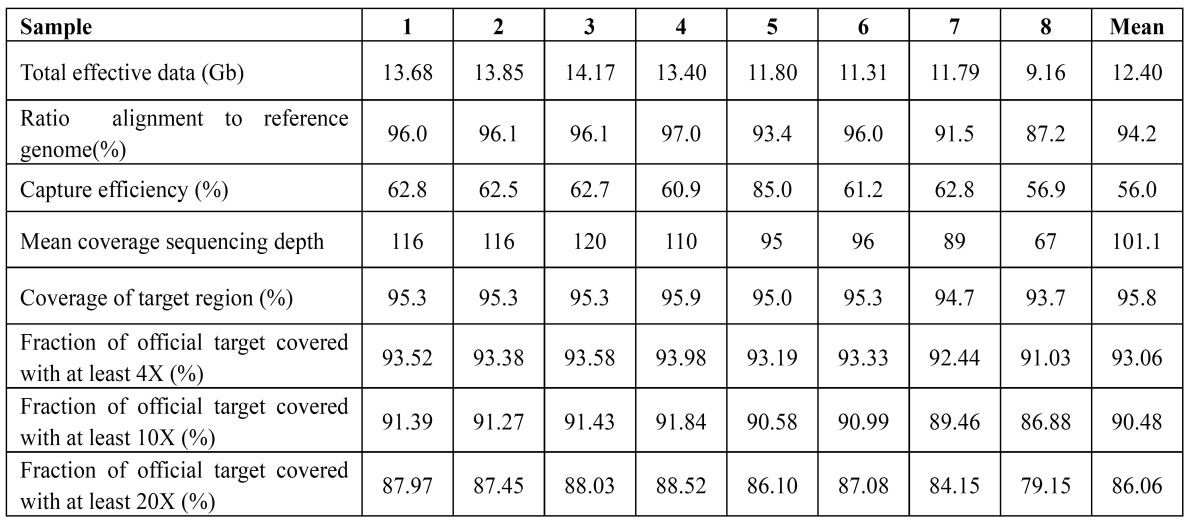
Quality evaluation of whole-exome sequencing.

An average of 138766 high-confidence single nucleotide variants (SNVs) per sample was found after the application of initial quality filtering criteria (coverage≥5X and SNP quality score≥40) following the manufacturer’s protocols. Then, we used the analytical procedures to significantly reduce the candidate list and focus on variants that were most likely to be relevant to NSCL/P. On average, a total 18759 SNVs per sample remained after filtering the SNVs not in exons. By filtering out synonymous variants in the coding region unlikely to be disease-causing variants, average 8575 exon non synonymous SNVs remained predicted to change an amino acid or a consensus splice site. The variant of IRF6, a known gene causing NSCL/P (12,14,25) , was included in these variants. Several other reported candidate genes including CRISPLD2, MYH9, PDGF-C and ABCA4 were also identified (16,26-28). NCBI dbSNP 135 and 1000 Genomes Project were utilized for filtering variants to remove presumably common variants. Each sequenced individual harbored an average 203 SNVs ranging form 167 to 231 ( Table 2).

**Table 2:**
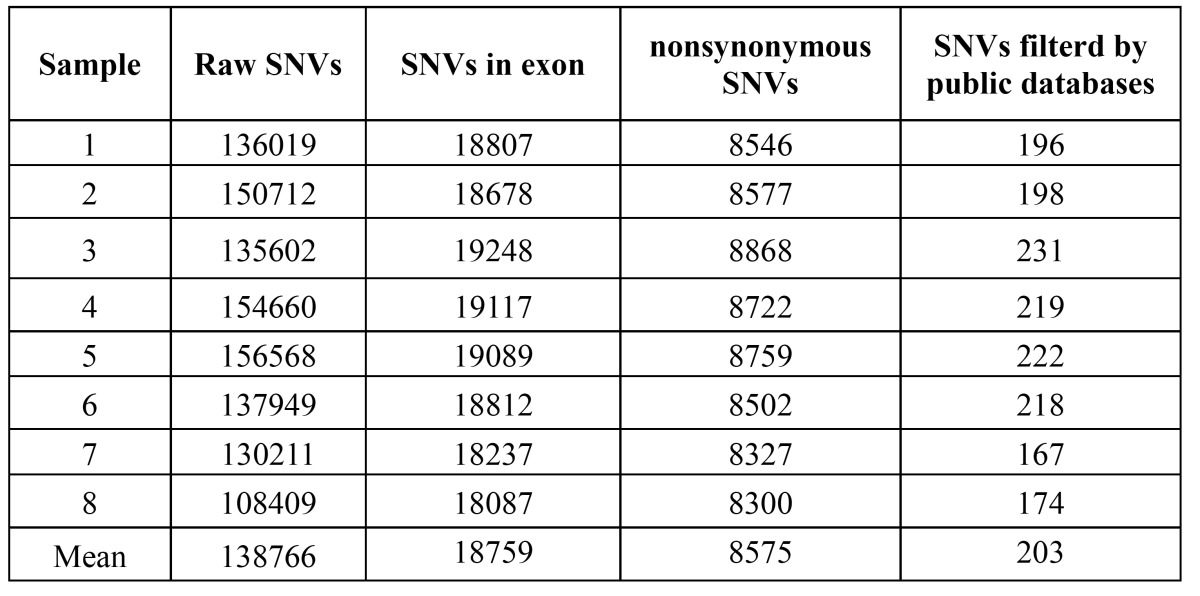
Summary of original whole-exome sequencing of SNVs data.

**Figure 2:**
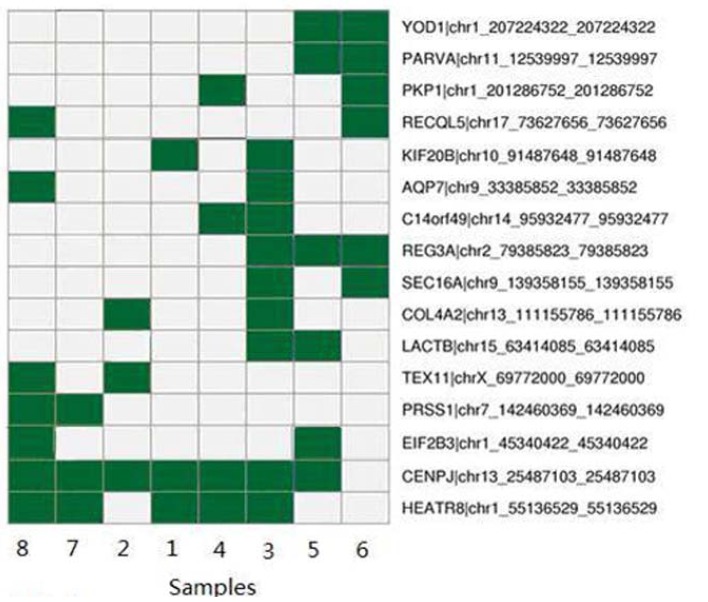
Heatmap of SNVs identified in 8 NSCL/P samples by whole-exome sequencing and Sanger sequencing. The DNA variants generated by whole-exome sequencing were verified by Sanger sequencing. The 16 types of SNVs were present in at least two samples. Therefore, whole-exome sequencing and Sanger sequencing confirmed 16 types of SNVs in at least 2 samples needed to be further studied.

In order to verify the DNA sequence variants detected by WES, the SNVs including some reported and novel genes through conventional Sanger sequencing method. In total, 12 reported candidate genes including IRF6, CRISPLD2, MYH9, PDGF-C and ABCA4 were detected by Sanger sequencing ( Table 3). Except for the reported genes, we found 16 novel SNVs were concordant with the gold standard method Sanger sequencing. The 16 SNVs were present in two or more samples ( Table 4, Fig. 2).

**Table 3:**
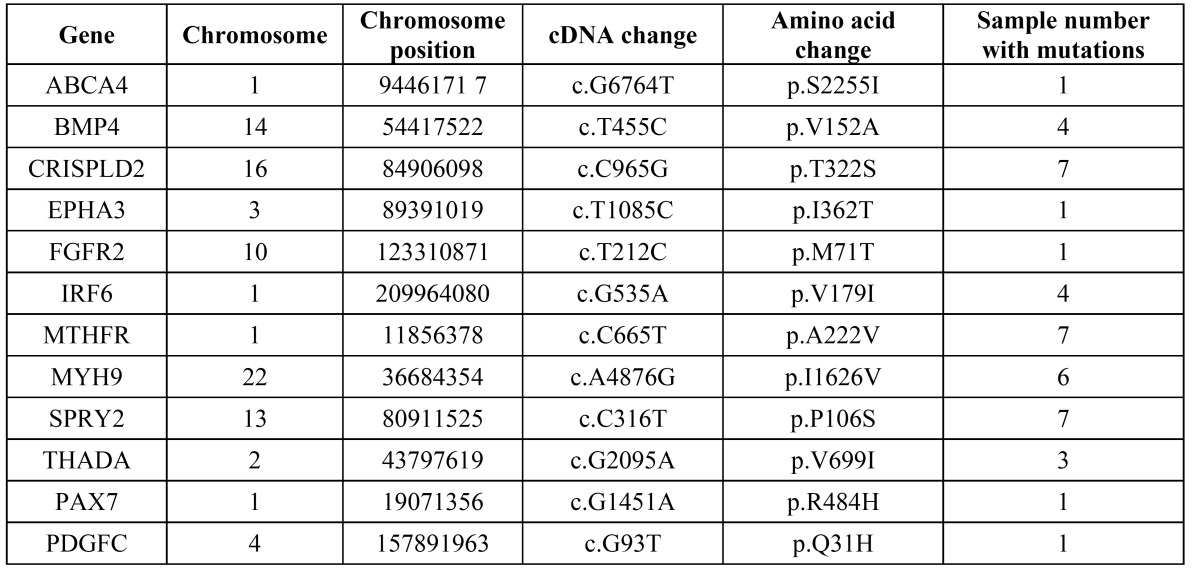
Reported SNVs identified in NSCL/P using whole-exome sequencing and Sanger sequencing.

**Table 4:**
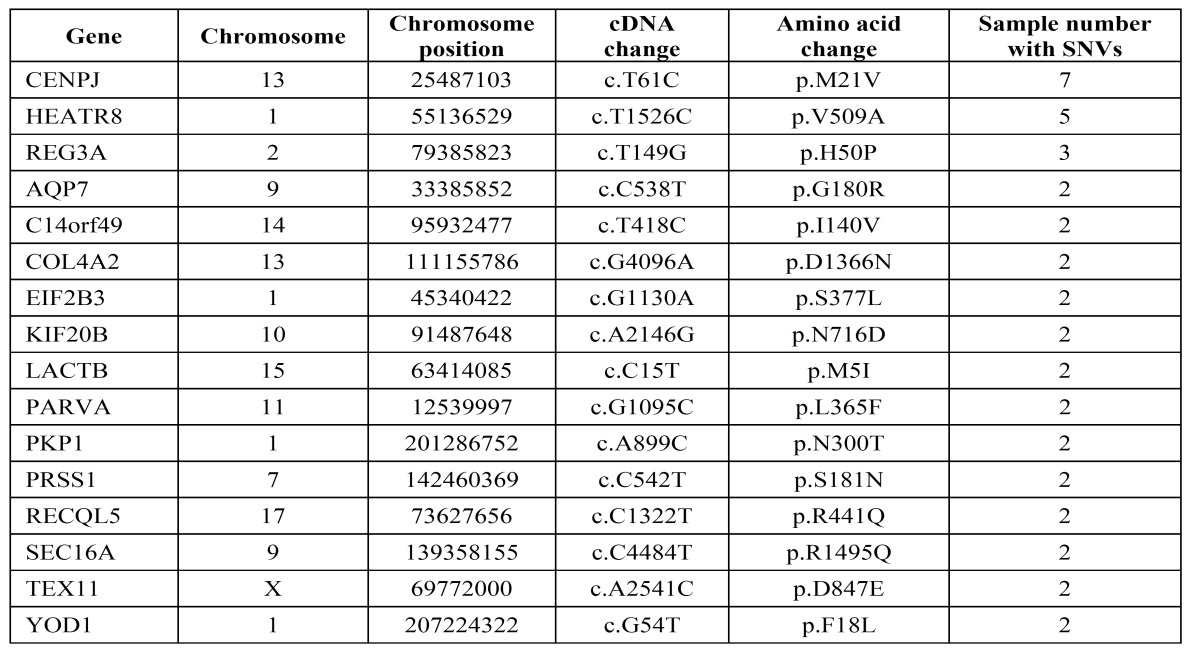
Novel SNVs identified in NSCL/P using whole-exome sequencing and Sanger sequencing.

## Discussion

NSCL/P is common craniofacial malformation with a complex and heterogeneous etiology. In the present study, we applied WES to search for the genetic variants of 8 samples with NSCL/P. We identified 16 novel SNVs shared by two or more affected samples which might play an important etiological role in NSCL/P. The study uncovered a list of susceptibility genes of NSCL/P and highlighted the important role of WES in identifying the etiology of NSCL/P.

Owing to its genetic heterogeneity and departure from Mendelian inheritance patterns, the identification of causative variants of NSCL/P has remained elusive. With the recent development of innovative approaches, whole-genome and WES projects become available now. WES has recently been successful in identifying causative genetic variants for Mendelian traits including Miller syndrome and Kabuki syndrome (22,23). In one recent study, the WES was used to study potentially causal variants in affected relatives drawn from multiplex cleft families (24). In our present study, we applied WES to identify genetic variants in Chinese subjects. We have shown WES may serve as a cost-effective and reproducible strategy for the identification of variants causing protein-coding changes in human genomes of NSCL/P. The average mean coverage sequencing depth on official target was 101.1-fold and with the average coverage of target region was 95.8%. Therefore, the coverage should have been adequate to reliably detect DNA variants within the majority of the targeted regions. After filtering the SNVs not included in exons, the average number of SNVs per sample was 18,759 and the number of non synonymous SNVs was 8,575. This study indicates that the current WES protocol can dissect the genetic etiology of NSCL/P. We speculate WES will be used in diagnosis of NSCL/P.

The susceptibility genes of NSCL/P are enormously diverse and complex screened by WES. The key challenge is how to identify the candidate genes from the large number of variants. In the present study, the average SNVs per sample were 18759 after filtering the SNVs not in exon. Even excluded the synonymous SNVs in the coding region, total 8575 exon non synonymous SNVs remained. We then applied NCBI dbSNP 135 and 1000 Genomes Project for filtering of variants to remove common variants. The availability of NCBI dbSNP 135 and 1000 Genomes Project is clearly helpful in generating a catalogue of common variation and necessary for filtering out them. Combining the two catalogues, the number of candidates reduced considerably and the susceptibility genetic variants list could be narrowed. Each sample carried 167 to 231 novel genetic variants with the average of 203.

In this study, the reported candidate genes were validated using Sanger sequencing. The results were consistent with some previous findings. The IRF6 variant known to be associated with NSCLP was also identified in these NSCLP-affected fetuses. In the studies of candidate genes for NSCL/P, many genes had positive results in one or more of the studies (5,11,13). However, only the positive association between IRF6 variants and NSCL/P has been confirmed in multiple populations of different ancestry (12,14,25). IRF6 is a key determinant of the keratinocyte proliferation and differentiation switch and also plays a key role in the formation of oral periderm, spatio-temporal regulation of which is essential in ensuring appropriate palatal adhesion (29). The studies confirmed IRF6 gene was one of the main candidate genes associated with NSCLP. With the exception of IRF6, many other candidate genes were present in the susceptibility genes identified by WES. CRISPLD2 is expressed in the craniofacial region during critical time points of palatal fusion. Genetic variation in CRISPLD2 has been shown to have a role in the etiology of NSCLP (27). MYH9, the gene coding for the heavy chain of non-muscle myosin II, has been considered as a good candidate gene in NSCL/P on the basis of its expression profile during craniofacial morphogenesis (26). The PDGF-C is essential for palatogenesis and is associated with NSCL/P (28). ABCA4 encodes an ATP-binding cassette transporter. A genome-wide association study of NSCL/P identifies risk variants near ABCA4 with stronger signals in Asian compared to European populations (16). The candidate genes associated with NSCL/P reported previously were identified in this study. The study suggests that direct sequencing of exons of small numbers of individuals can serve as a useful approach for identifying genetic variants of NSCL/P. As the costs of WES become more reasonable, we believe the project can be useful in clinical diagnosis of NSCL/P.

However, this study failed to identify some reported candidate genes including FOXE1, NOG and VAX1. The gene FOXE1 variants involve all combinations of primary and secondary palatal clefts and have a significant role in the etiology of NSCL/P (30). Mangold and colleagues identified two candidate genes NOG and VAX1 were associated with NSCL/P in populations from European ancestry (19). The failure to detect the association of the genes FOXE1, NOG and VAX1 with NSCL/P may due to small size of the study or genetic heterogeneity.

In this study, Sanger sequencing was further applied to validate some novel genes. In total, 16 SNVs were present in two or more samples. The 16 SNVs may be predicted to lead to a significantly changed protein product. Then, the 16 non synonymous variants shared by two or more samples are highly likely to be causative for NSCL/P. Therefore, WES and Sanger sequencing confirmed 16 different types of prioritized SNVs associated with NSCL/P. Our study presented a preliminary overview of the genetic etiology of NSCL/P. The SNVs identified in the study may play important roles in susceptibility to NSCL/P. Although we were unable to confirm rigorously whether any of these genes indeed contributed to NSCL/P, our results provided a prioritized shortlist for further association and validation studies. We speculate the susceptibility genes identified by WES may also contribute in some way to NSCL/P. These new candidates are needed be evaluated in future studies to establish their true relevance to NSCL/P susceptibility. Although the exact biological mechanism of the susceptibility genes involved in NSCL/P pathogenesis still needs to be clarified, genetic findings in this study will provide helps in diagnosis, counseling and treatment of NSCL/P.

In this study, we have demonstrated WES represents a cost-effective, reproducible and robust approach for revealing the etiology of NSCL/P. We have successfully applied WES for genetic variants screening of NSCL/P. Our study has generated a list of susceptibility genes of NSCL/P to lead to a full understanding of NSCL/P and improved information and clinical applications for affected individuals. The susceptibility genes identified in fetuses affected by NSCLP may lead to improved diagnosis, counseling and treatment.

## References

[1] Mossey PA, Little J, Munger RG, Dixon MJ, Shaw WC (2009). Cleft lip and palate. Lancet.

[2] Ngai CW, Martin WL, Tonks A, Wyldes MP, Kilby MD (2005). Are isolated facial cleft lip and palate associated with increased perinatal mortality? A cohort study from the West Midlands Region, 1995-1997. J Matern Fetal Neonatal Med.

[3] Marazita ML (2012). The evolution of human genetic studies of cleft lip and cleft palate. Annu Rev G nomics Hum Genet.

[4] Wu T, Liang KY, Hetmanski JB, Ruczinski I, Fallin MD, Ingersoll RG (2010). Evidence of gene environment interaction for the IRF6 gene and maternal multivitamin supplementation in contro ling the risk of cleft lip with/without cleft palate. Hum Genet.

[5] Stuppia L, Capogreco M, Marzo G, La Rovere D, Antonucci I, Gatta V (2011). Genetics of sy dromic and nonsyndromic cleft lip and palate. J Craniofac Surg.

[6] Rahimov F, Jugessur A, Murray JC (2012). Genetics of nonsyndromic orofacial clefts. Cleft Palate Cr niofac J.

[7] Jugessur A, Farlie PG, Kilpatrick N (2009). The genetics of isolated orofacial clefts: from genotypes to subphenotypes. Oral Dis.

[8] Park JW, Cai J, McIntosh I, Jabs EW, Fallin MD, Ingersoll R (2006). High throughput SNP and expression analyses of candidate genes for non-syndromic oral clefts. J Med Genet.

[9] Dixon MJ, Marazita ML, Beaty TH, Murray JC (2011). Cleft lip and palate: understanding genetic and environmental influences. Nat Rev Genet.

[10] Ingersoll RG, Hetmanski J, Park JW, Fallin MD, McIntosh I, Wu-Chou YH (2010). Association between genes on chromosome 4p16 and non-syndromic oral clefts in four populations. Eur J Hum Genet.

[11] Marazita ML, Murray JC, Lidral AC, Arcos-Burgos M, Cooper ME, Goldstein T (2004). Meta analysis of 13 genome scans reveals multiple cleft lip/palate genes with novel loci on 9q21 an2q32 35. Am J Hum Genet.

[12] Park JW, McIntosh I, Hetmanski JB, Jabs EW, Vander KCA, Wu-Chou YH (2007). Association between IRF6 and nonsyndromic cleft lip with or without cleft palate in four populations. Genet Med.

[13] Vieira AR (2006). Association between the transforming growth factor alpha gene and nonsyndromic oral clefts: a HuGE review. Am J Epidemiol.

[14] Zucchero TM, Cooper ME, Maher BS, Daack-Hirsch S, Nepomuceno B, Ribeiro L (2004). Inte feron regulatory factor 6 (IRF6) gene variants and the risk of isolated cleft lip or palate. N Engl J Med.

[15] Kahvejian A, Quackenbush J, Thompson JF (2008). What would you do if you could sequence ever thing. Nat Biotechnol.

[16] Beaty TH, Murray JC, Marazita ML, Munger RG, Ruczinski I, Hetmanski JB (2010). A genome wide association study of cleft lip with and without cleft palate identifies risk variants near MAFB and ABCA4. Nat Genet.

[17] Birnbaum S, Ludwig KU, Reutter H, Herms S, Steffens M, Rubini M (2009). Key susceptibility locus for nonsyndromic cleft lip with or without cleft palate on chromosome 8q24. Nat Genet.

[18] Grant SF, Wang K, Zhang H, Glaberson W, Annaiah K, Kim CE (2009). A genome-wide associ tion study identifies a locus for nonsyndromic cleft lip with or without cleft palate on 8q24. J Ped atr.

[19] Mangold E, Ludwig KU, Birnbaum S, Baluardo C, Ferrian M, Herms S (2010). Genome-wide association study identifies two susceptibility loci for nonsyndromic cleft lip with or without cleft palate. Nat Genet.

[20] Ng SB, Turner EH, Robertson PD, Flygare SD, Bigham AW, Lee C (2009). Targeted capture and massively parallel sequencing of 12 human exomes. Nature.

[21] Gonzaga-Jauregui C, Lupski JR, Gibbs RA (2012). Human genome sequencing in health and disease. Annu Rev Med.

[22] Ng SB, Buckingham KJ, Lee C, Bigham AW, Tabor HK, Dent KM (2010). Exome sequencing identifies the cause of a mendelian disorder. Nat Genet.

[23] Norton N, Li D, Rieder MJ, Siegfried JD, Rampersaud E, Zuchner S (2011). Genome-wide stu ies of copy number variation and exome sequencing identify rare variants in BAG3 as a cause of dilated cardiomyopathy. Am J Hum Genet.

[24] Bureau A, Parker MM, Ruczinski I, Taub MA, Marazita ML, Murray JC (2014). Whole Exome Sequencing of Distant Relatives in Multiplex Families Implicates Rare Variants in Candidate Genes for Oral Clefts. Genetics.

[25] Rahimov F, Marazita ML, Visel A, Cooper ME, Hitchler MJ, Rubini M (2008). Disruption of an AP-2alpha binding site in an IRF6 enhancer is associated with cleft lip. Nat Genet.

[26] Birnbaum S, Reutter H, Mende M, de Assis NA, Diaz-Lacava A, Herms S (2009). Further ev dence for the involvement of MYH9 in the etiology of non-syndromic cleft lip with or without cleft palate. Eur J Oral Sci.

[27] Chiquet BT, Lidral AC, Stal S, Mulliken JB, Moreno LM, Arcos-Burgos M (2007). CRISPLD2: a novel NSCLP candidate gene. Hum Mol Genet.

[28] Choi SJ, Marazita ML, Hart PS, Sulima PP, Field LL, McHenry TG (2009). The PDGF-C regula ory region SNP rs28999109 decreases promoter transcriptional activity and is associated with CL/P. Eur J Hum Genet.

[29] Richardson RJ, Dixon J, Jiang R, Dixon MJ (2009). Integration of IRF6 and Jagged2 signalling is esential for controlling palatal adhesion and fusion competence. Hum Mol Genet.

[30] Moreno LM, Mansilla MA, Bullard SA, Cooper ME, Busch TD, Machida J (2009). FOXE1 ass ciation with both isolated cleft lip with or without cleft palate, and isolated cleft palate. Hum Mol Genet.

